# Special Issue “Drug Treatments for Inflammatory Bowel Diseases”

**DOI:** 10.3390/ph17010059

**Published:** 2023-12-29

**Authors:** Anderson Luiz-Ferreira, Carmine Stolfi

**Affiliations:** 1 Inflammatory Bowel Disease Research Laboratory, Department of Biological Sciences, Institute of Biotechnology, Federal University of Catalão (UFCAT), Catalão 75704-020, GO, Brazil; 2 Department of Systems Medicine, University of Rome “Tor Vergata”, 00133 Rome, Italy; carmine.stolfi@uniroma2.it

Inflammatory bowel diseases (IBD), including Crohn’s disease (CD) and ulcerative colitis (UC), are chronic idiopathic, relapsing and remitting inflammatory diseases that affect the gastrointestinal tract, causing significant morbidity and loss of quality of life in affected individuals [1]. In UC, inflammation is mainly restricted to the mucosa and involves only the colon, although the extent of colic involvement may vary among patients and over time [2]. In CD, the inflammatory process is transmural and affects any portion of the gastrointestinal tract, from the mouth to the anus, and may be associated with penetrating phenomena such as intraperitoneal abscesses or fistulas [3]. Although the etiology is unknown, it is believed that the interaction between environmental factors and the gut microbiota of a genetically susceptible host leads to the abnormal immune response of the colic mucosa observed in these diseases [4].

Traditionally, the process of drug development for the treatment of IBD involves the discovery and selection of targets, followed by biological confirmation in cellular and animal models. When promising results are found at this stage, phase I, II and III clinical studies are carried out to investigate the safety, pharmacokinetics, clinical efficacy, and dosage of the drug [5].

Conventional therapies such as aminosalicylic acid, corticosteroids, immunomodulators and anti-tumor necrosis factor agents continue to demonstrate therapeutic efficacy, particularly when used in combination. However, despite the variety of therapeutic compounds available and the improved management strategies, a portion of IBD patients with moderate to severe degrees of the disease do not benefit from the existing treatments or experience side effects caused by the drugs used [6,7]. This has been a driving force for the intensive search for new drugs aimed at IBD therapy as well as the evaluation of potential IBD-related adverse effects [7].

In fact, aiming to increase the range of therapeutic options, new strategies with other biological agents and, more recently, with small-molecule drugs have been explored. Based on more accurate and targeted management of the disease, treatments with fewer adverse effects have been sought. In recent efforts, new inhibitors, whose targets include cytokines (such as IL-12/23 inhibitors, PDE4 inhibitors) [8,9], integrins (such as integrin inhibitors) [10], cytokine signaling pathways (such as Janus Kinases—JAK inhibitors) [11], and cell signaling receptors (such as Sphingosine 1-Phosphate Receptor—S1p modulators) [12], have become potentially promising therapeutic choices for many IBD patients.

Computational methods have also been employed in the development of drugs for the treatment of IBD, aiming to discover drugs in a more sustainable and economical way. These methods, together with the traditional drug development process, have contributed not only to the identification of more specific therapeutic targets, but also to novel applications of existing drugs [13].

This Special Issue included eight current research articles and three review articles with contributions to therapeutic decision-making and propositions of new therapeutic targets for IBD. We emphasize that it is not the objective of this Editorial to make a detailed description of each of the works, but rather to encourage the reader to explore them.

Two research studies published in this Special Issue aimed to investigate how two antidiabetic drugs of the thiazolidinedione class of agonists of the nuclear hormone receptor ‘peroxisome proliferator-activated receptor gamma’ (PPARγ), rosiglitazone and pioglitazone, can affect the risk of IBD.

In the first of these studies, Tseng (contribution 1) demonstrated that the use of rosiglitazone does not affect IBD risk, based on information from the reimbursement database of Taiwan’s National Health Insurance. However, as this was an observational study with a small number of cases of UC, it was not possible to exclude the benefit of rosiglitazone for patients affected specifically by this pathology. In the second study, using the same database, Tseng further observed that pioglitazone had no effect on IBD at the doses used for the Taiwanese population (contribution 2).

With the objective of improving disease management strategies, two articles published in this Special Issue addressed strategies that can, in some way, assist in therapeutic decision-making: one related to the gene expression of mediators in the colic mucosa and the other related to changes in the form of drug administration.

Alafarj et al. (contribution 3) investigated changes in gene expression in the colic mucosa of IBD patients treated with 5-amino salicylic acid or biological therapy (anti-TNF drugs), IBD patients receiving no medication, and individuals without IBD. The study showed the importance of molecular analysis of biomarkers in the evaluation of inflammation, contributing to therapeutic decision-making.

Oršić Frič et al. (contribution 4) compared the vedolizumab serum trough concentration, efficacy and safety before and six months after changing the route of administration from intravenous to subcutaneous, but in a low number of patients. The authors demonstrated that the mean trough serum concentration of subcutaneous vedolizumab was significantly higher than that of intravenous vedolizumab, with the efficacy and safety previously established for patients on maintenance therapy with intravenous vedolizumab.

Four articles addressed the search for therapeutic alternatives for the treatment of IBD (contributions 5 to 8).

Lebish et al. (contribution 5) used inhibitors of the MAPK-activated protein kinase 2 (MK2) pathway, an important pathway in the regulation of cytokines in IBD and highly expressed and activated in tissues of patients affected by CD, to study a new therapeutic alternative. It was identified that MK2 regulates the expression of specific matrix metalloproteinases, and its inhibition decreases not only T-cell activity, but also the production of inflammatory cytokines. In view of previously published data on the safety of MK2 inhibitors in humans and the results presented in this study, Lebish et al. suggested that MK2 could be a new therapeutic target for CD.

Saber et al. (contribution 6) introduced a new therapeutic approach in order to improve the treatment of chronic UC. In this study, (R,R)-BD-AcAc2, a type of ketone ester (KE), improved the macroscopic and microscopic characteristics of the colon, exhibiting anti-inflammatory properties by reducing the production of pro-inflammatory cytokines. Although the use of an animal model cannot fully capture the intricate complexities of human pathophysiology, the authors concluded that this preclinical study indicated a potential therapeutic benefit of ketosis and ketone production in the treatment of IBD.

Assuming that the transforming growth factor-β/Smad (TGF-β/Smad) signaling pathway is inactivated in CD patients by Smad 7 overexpression and that clinical studies using oral Smad 7 antisense oligonucleotides were discontinued, Tsujimura et al. (contribution 7) tested the complex formed by microRNA (miR-497a-5p) and apatite super carbonate nanoparticle (sCA-miR-497a-5p) in the DSS-induced colitis model. Through the regulation of multiple genes rather than a single molecule, sCA-miR-497a-5p exerted a potent anti-inflammatory effect through the activation of the TGF-β/Smad signaling pathway and the inhibition of secretion inflammatory cytokines. Therefore, the authors suggested that this complex may have a therapeutic ability against IBD.

Jin et al. (contribution 8) investigated the activity of Ren-Shen-Bai-Du powder (RSBDP), which is already currently used for the treatment of IBD in China. RSBDP protected the colic mucosa of DSS-challenged animals by reducing the concentration of pro-inflammatory cytokines and promoting apoptosis of intestinal epithelial cells. The authors attributed the pharmacological effects shown by RSBDP to the main specialized metabolites (quercetin, kaempferol, luteolin, naringenin, and sitosterol) present in its constitution and provided contributions regarding the anti-inflammatory mechanisms of the currently used RSBDP in IBD therapy.

In addition to research articles, three interesting review articles were published in this Special Issue.

In the first, Blagov et al. (contribution 9) conducted a review of studies published with experimental models and clinical studies and described in detail the involvement of oxidative stress, with the accumulation of reactive oxygen species (ROS), in the chronic inflammation observed in IBD. The effects and mechanism of action of several natural antioxidants, as well as other antioxidants that have not yet been tested in the treatment of IBD, were also considered in this work.

The second review article (contribution 10) assembled the major targets and agents currently directed at the production of pro-inflammatory cytokines and lymphatic trafficking in order to contribute to the development of new drugs for IBD.

Finally, Imbrizi et al. (contribution 11) carried out a narrative review that elegantly presented the therapeutic advances in the treatment of IBD, the mechanisms of action, and the challenges facing the therapeutic goals in the treatment of IBD. Interestingly, the authors showed that despite the different mechanisms of action of the different classes of drugs, the general rates of effectiveness are similar, including the latest therapeutic classes approved for the treatment of IBD, JAK inhibitors and S1p modulators.

In conclusion, conventional therapies are still widely used, especially in the treatment of mild and moderate levels of IBD. However, the variable responses of IBD patients can lead to relapses, and this highlights the need to explore new treatment alternatives capable of addressing unmet needs and reducing adverse effects. The development of new therapeutic classes, as well as the combination of biological agents and small molecules, can bring substantial benefits for the therapeutic management of IBD. Finally, computational approaches have been used to identify metabolite–target interactions, providing several new drug targets for potential immune therapies.

We hope that the articles and reviews in this Special Issue meet the expectations of readers in the field and further promote investigations on the treatment of IBD by the scientific community.

## Data Availability

Data is contained within the article.
